# Ethical and academic factors influencing the moral courage of nursing students

**DOI:** 10.1590/0034-7167-2025-0389

**Published:** 2026-08-21

**Authors:** Romario Daniel Jantara, Jamila Geri Tomaschewski Barlem, Danubia Andressa da Silva Stigger, Sabrina Santos da Rocha, Rhaissa Grazielli Vieira de Azevêdo, Edison Luiz Devos Barlem, Tatiane Baratieri, Kátia Pereira de Borba

**Affiliations:** I Universidade Estadual do Centro-Oeste. Guarapuava, Paraná, Brazil; II Universidade Federal do Rio Grande. Rio Grande, Rio Grande do Sul, Brazil

**Keywords:** Courage, Morale, Nursing Ethics, Nursing, Nursing Students., Coraje, Moral, Ética en Enfermería, Enfermería, Estudiantes de Enfermería.

## Abstract

**Objectives:**

to assess the levels of moral courage among Brazilian undergraduate nursing students and examine the ethical and academic factors that influence it.

**Methods:**

cross-sectional study conducted with 130 nursing students who answered an online questionnaire containing sociodemographic information, variables related to the ethical and academic context, and the Brazilian version of the Nurses’ Moral Courage Scale Descriptive statistics, t-tests, ANOVA, Spearman’s correlation, and multiple linear regression were performed.

**Results:**

the average moral courage score was 61.58 (±9.55), positively correlated with satisfaction with the program, academic performance, relationship with educators, and especially confidence in one’s own ethical principles. The strongest predictors were confidence in ethical principles (β = 0.455) and academic performance (β = 0.225), explaining 26.9% of the variance.

**Conclusions:**

findings suggest that fostering students’ ethical confidence and academic success may strengthen their moral courage, with implications for nursing education and professional development.

## RESUMEN

**Objetivos:**

evaluar los niveles de coraje moral entre estudiantes de graduación en enfermería brasileños y examinar los factores éticos y académicos que la influencian.

**Métodos:**

estudio transversal con 130 estudiantes de enfermería, quienes respondieron un cuestionario en línea que incluyó información sociodemográfica, variables de contexto ético y académico y la versión brasileña de la Nurses’ Moral Courage Scale. Se emplearon análisis descriptivos, pruebas t, ANOVA, correlación de Spearman y regresión lineal múltiple.

**Resultados:**

la puntuación de coraje moral fue de 61,58 (±9,55), mostrando correlación positiva con la satisfacción con el curso, el rendimiento académico, la relación con los docentes y la confianza en los principios éticos. Los predictores más fuertes fueron la confianza en los principios éticos (β = 0,455) y el rendimiento académico (β = 0,225), explicando el 26,9% de la varianza.

**Conclusiones:**

fomentar la confianza ética y el éxito académico puede fortalecer el coraje moral.

## INTRODUCTION

Healthcare professionals encounter complex ethical challenges that contribute to burnout and moral distress^(1)^. Moral distress is a globally recognized issue that significantly impacts healthcare workers^(2)^. Among these workers, nurses play a central role in the continuum of health and disease, providing care across the population’s life cycle. This responsibility requires these workers to address the needs of individuals with diverse pathologies and injuries, exposing nurses to considerable stressors. Such scenarios frequently involve ethical dilemmas that demand effective responses guided by ethical and moral principles^(3)^.

Moral challenges influence the development of nurses’ professional identity and ethical commitment, as identity is shaped by self-perception, professional role, interpersonal relationships, work organization, and experiences^(4)^. Research indicates that nurses often recognize the appropriate course of action but are frequently unable to act accordingly^(5)^. Therefore, nurses must develop moral competencies to address these difficulties to provide high-quality care and mitigate moral distress^(6)^.

Nursing students are immersed in this context and will soon encounter ethically challenging situations and moral distress in their professional practice. Hence, they must be ethically informed and morally sensitive to handle such situations effectively, ensuring holistic care guided by strong ethical decision-making skills. Unethical behavior among nursing students can undermine their learning and training, leading to unprofessional conduct, negligence, weakened patient relationships, substandard nursing care, and increased risks to patient safety^(7)^.

In this context, nursing is recognized as an ethical practice that demands the courage to uphold moral principles, advocate for what is right, and act according to one’s values. Moral courage is essential for nurses across all areas and levels of health care. The renewed focus on virtue ethics in health care has contributed to a growing recognition of moral courage as both a virtue and a fundamental component of human morality^(8)^.

Moral courage refers to an individual’s inner strength to navigate ethical conflicts following ethical principles, personal values, and beliefs, even when doing so may result in adverse consequences^(9)^. It is a virtue that requires deliberation and ethical action and is key to nurses’ overall moral competence. Deeply connected to the professional values of nursing, moral courage is essential for upholding these principles in daily practice. Rather than being limited to heroic acts by exceptional nurses in extraordinary situations, it is a complex and fundamental concept in ethical nursing care^(8)^.

Moral courage has gained increasing attention as a key component of nurses’ moral competence, underscoring the need to identify the factors that influence it to enhance the quality of nursing care. As a virtue that can be taught, learned, and practiced, understanding its determinants from both organizational and personal perspectives is essential for further strengthening moral courage^(10)^.

A scoping review identified several factors associated with moral courage in nursing students, including moral distress, moral sensitivity, age, and prior training in the health field, among others. The review also suggested that moral courage may be a potential predictor of anxiety disorders, acute stress disorder, and other mental health conditions; however, the need for further research it emphasized to confirm these findings^(11)^. Moreover, the literature has not provided systematic and consistent evidence on the ethical and academic factors influencing nursing students’ moral courage, which is a critical step toward developing effective interventions to strengthen moral courage within this population.

## OBJECTIVES

To assess the levels of moral courage among Brazilian undergraduate nursing students and examine the ethical and academic factors that influence it.

## METHODS

### Ethical considerations

This study adhered to ethical principles and complied with Resolution No. 510 of April 7, 2016, issued by the Brazilian National Health Council. It is part of a larger project funded by National Council for Scientific and Technological Development (CNPq), titled “*Sofrimento Moral, Resiliência Moral e Coragem Moral na formação e prática de enfermeiros*”. The Institutional Review Board approved the study under opinion No. 6.034.053. A free and informed consent form was presented on the first page of the form, emphasizing the confidentiality of participants’ information, the voluntary nature of participation, the right to withdraw from the study at any time, and the provision of medical assistance in case of any discomfort caused by participation.

### Study Design, Period, and Location

This quantitative study employed a cross-sectional, descriptive, and correlational design. The study was conducted in accordance with the STROBE (Strengthening the Reporting of Observational Studies in Epidemiology) guidelines.

Data were collected between April and September 2024 using an electronic form hosted on Google Forms. Authorization was obtained from the coordinators and professors of the undergraduate nursing program to invite students to participate either during class or via WhatsApp/email. Participants were informed about the study’s objectives, assured of the confidentiality of their information, and made aware of their right to withdraw at any time. A link to the electronic form was provided, which became accessible after participants signed a free and informed consent form available on the first page. An electronic copy of the form and their responses was automatically sent to the participants’ registered email. Whenever possible, class time was allocated for students to complete the questionnaire, which took approximately 20 minutes.

### Population and Sample; Inclusion and Exclusion Criteria

Non-probabilistic convenience sampling was used, with the sample size calculated using the StatCalc module of Epi Info 7.2.5.0^(12)^. The inclusion criterion required participants to be regularly enrolled from the third semester onward, as practical clinical experience was necessary to complete the questionnaire. Among the 186 nursing students enrolled from the third semester onward at the host institution, the minimum required sample size was determined to be 125 students based on a 95% confidence level. Ultimately, 130 nursing students completed the study form without withdrawals or missing responses.

### Study Protocol

A sociodemographic questionnaire was developed to characterize ethical and academic context variables, and the Brazilian Nurses’ Moral Courage Scale (NMCS)^(13)^, was adopted.

The sociodemographic questionnaire and the characterization of ethical and academic context variables were divided into two sections: (1) Sociodemographic Data and (2) Professional and Academic Background. The form comprised 33 items to collect social, sociodemographic, and contextual variables that may influence moral courage, as identified in the international literature(11). Marital status, ethnicity, family income, religion, and program semester were answered using predefined options. Age was measured in years, while gender was categorized dichotomously (male/female). Additionally, the following variables were assessed as dichotomous (yes/no): prior training in the health field, witnessing a morally incorrect or unethical situation involving a peer, and active engagement in an ethics-related task (e.g., research group).

The ethical and academic context variables included satisfaction with the program, self-assessment of academic performance, evaluation of relationships with educators, assessment of ethics teaching within the program, and confidence in one’s own ethical principles. These items were rated on a Likert scale, and PhDs with expertise in nursing ethics reviewed the instrument.

The Nurses’ Moral Courage Scale (NMCS) was originally developed in Finland. Initially, it comprised 21 items across four dimensions: (1) compassion and true presence, (2) moral responsibility, (3) moral integrity, and (4) commitment to good care. Responses are rated on a 5-point Likert scale, where respondents indicate how well each item describes them, ranging from 1 (does not describe me at all) to 5 (describes me very well). Higher scores reflect greater self-assessed moral courage^(14)^.

The scale was validated in the Brazilian context for use with nursing students, adopting a unidimensional structure with 15 items and a scoring range from 15 to 75. The scale demonstrated strong reliability indices, with the Greatest Lower Bound to Reliability at 1.00, McDonald’s Omega at 0.91, and Cronbach’s Alpha at 0.91^(13)^.

### Data Analysis and Statistics

Data were tabulated in Microsoft Excel and analyzed quantitatively using descriptive and inferential statistics. The descriptive analysis included calculations of frequencies, means, standard deviations, skewness, and kurtosis. Inferential statistics comprised Student’s t-test, one-way analysis of variance (ANOVA), Spearman correlation, and multiple linear regression analysis. The Statistical Package for the Social Sciences (SPSS), version 20.0, was used in all analyses.

The normality of the Moral Courage scores was assessed using the Kolmogorov-Smirnov test with Lilliefors correction and the Shapiro-Wilk test. Results indicated that the Moral Courage variable was not normally distributed (K-S(130) = 0.094, p = 0.006; S-W(130) = 0.906, p < 0.001), and the same pattern was observed for the other variables.

ANOVA was conducted to examine differences in moral courage means across marital status, program semester, age group, family income, ethnicity, and religion. The Student’s t-test was used to compare moral courage means between groups based on gender, failure in a subject, prior training in the health field, witnessing a morally incorrect or unethical event, and active engagement in ethics-related tasks. Additionally, the t-test compared moral courage means between students attending from the third to the sixth semester and those attending from the seventh to tenth semester, as well as between students with and without religion.

Spearman correlation was used to examine the relationship between moral courage and ethical and academic context variables, including satisfaction with the program, academic performance, relationships with educators, evaluation of ethics teaching, and confidence in one’s own ethical principles. A multiple linear regression analysis (enter method) was conducted to identify predictors of moral courage, satisfaction with the program, academic performance, relationships with educators, and confidence in one’s own ethical principles as independent variables.

Homogeneity of variance was assessed using Levene’s test to guide the interpretation of statistical tests. Bootstrapping with 1,000 resamplings and a 95% BCa CI (bias-corrected and accelerated bootstrap confidence interval) was applied to address deviations from normality and enhance result reliability. This procedure ensures that if the study were replicated 100 times, the results would fall within the margin of error in 95% of cases^(15)^.

## RESULTS

### Sociodemographic Characterization

Most of the 130 participants were women (86.2%), with an average age of 24.64 years (SD = 5.19; range: 19-48). The majority were single (84.6%), self-identified as White (74.6%), had a family income between one and two times the minimum wage (44.6%), and reported having no religion (38.5%). Regarding program semesters, 16.9% were in the third semester, 14.6% in the fourth, 16.2% in the fifth, 10.8% in the sixth, 16.9% in the seventh, 8.5% in the eighth, 14.6% in the ninth, and 1.5% in the tenth semester.

### Descriptive statistics of moral courage and other variables

The total score ranged from 17 to 75 points, with a mean of 61.58 and a standard deviation of 9.55. Table 1 presents the descriptive results of the remaining variables.

**Table 1:** Descriptive results concerning the program semester, ethics teaching, and moral distress among nursing students (N=130), Rio Grande, Rio Grande do Sul, Brazil, 2024

Variables		N=130	Percentage (%)
Program semester	3^rd^ semester	22	16.9
	4^th^ semester	19	14.6
	5^th^ semester	21	16.2
	6^th^ semester	14	10.8
	7^th^ semester	22	16.9
	8^th^ semester	11	8.5
	9^th^ semester	19	14.6
	10^th^ semester	2	1.5
Failure	Yes	45	34.6
	No	85	65.4
Previous degree in the health field	Yes	23	17.7
	No	107	82.3
Witnessed a morally incorrect or unethical event involved a classmate	Yes	60	46.2
	No	70	53.8
Actively engaged in a ethics-related task (e.g., research group)	Yes	12	9.2
	No	118	90.8
The student often feels that s/he should act with moral courage	Mean (SD^*^)		4.19 (SD=0.93)
Level of satisfaction with the program	Mean (SD^*^)		3.63 (SD= 0.83)
Self-assessed academic performance	Mean (SD^*^)		3.74 (SD= 0.74)
Relationship with educators	Mean (SD^*^)		3.82 (SD= 0.75)
Ethics teaching	Mean (SD^*^)		3.59 (SD=1.00)
Confidence in one’s own ethical principles	Mean (SD^*^)		4.20 (SD=0.76)

Differences in mean levels of moral courage according to the Sample sociodemographic characteristics

ANOVA corrected for data without homogeneity of variance (Levene(2, 127) = 3.171, *p* = 0.045) did not identify significant differences in moral courage means among students who were single, married, or in a stable union (Welch’s *F*(2, 12.034) = 0.353, *p* = 0.710). Similarly, no statistically significant difference was found across program semesters (*F* (7, 122) = 1.511, *p* = 0.170) (Levene(7, 122) = 0.568, *p* = 0.780). However, the Student’s *t*-test revealed that when students were grouped into those attending from the third to the sixth semester and those from the seventh to the tenth semester, the latter scored higher in moral courage (Table 2). A small effect size was found (Cohen’s *d* = 0.37).

**Table 2:** Results of the test of difference in levels of moral courage between the groups of semesters *t*-test (Bootstrapping sample) (N=130), Rio Grande, Rio Grande do Sul, Brazil, 2024

		*M*	*SD*	*t*	*df*	Mean difference	CI of the Mean Difference (95%)	
							Lower limit	Upper limit
Moral Courage	3^rd^ to the 6^th^	60.15	10.03	-2.08^^*^^	128	-3.50	57.98	62.26
	7^th^ to 10^th^	63.66	8.48				61.34	65.81

M - mean; SD - standard deviation;

* p - 0.039; df - degrees of freedom; CI - confidence interval.

ANOVA also showed no statistically significant difference between the levels of moral courage of students in different ages groups (between 20 and 24 years old, 25 and 29 years old, and over 29 years old) (Levene (2, 127) = 0.496, *p* = 0.496; *F* (2, 127) = 0.460, *p* = 0.632), different family incomes (Levene (3, 126) = 1.705, *p* = 0.169; *F* (3, 126) = 0.621, *p* = 0.603), ethnicity (Levene (3, 126) = 0.530, *p* = 0.663; *F* (3, 126) = 0.819, *p* = 0.486), or between different religions and no religion (Levene (4, 125) = 0.611, *p* = 0.655; *F* (4, 125) = 1.518, *p* = 0.201). However, when isolated into two groups, Student’s t-test showed that students with a religion exhibited a statistically higher level of moral courage (M = 62.97; SD = 8.84) than those without a religion (M = 59.36; SD = 10.29) (*t*(128) = -2.127, *p* = 0.035).

No significant differences were found in mean moral courage scores based on gender (*t*(128) = 0.917, *p* = 0.361), program failure (*t*(128) = 1.114, *p* = 0.267), previous training in the health field (*t*(128) = -0.562, *p* = 0.575), or active engagement in an ethics-related task (*t*(128) = 0.410, *p* = 0.682). However, students who had witnessed a morally incorrect or unethical situation scored significantly higher in moral courage (M = 64.11, SD = 7.72) than those who had not (M = 59.41, SD = 10.45) (*t*(128) = 2.874, *p* = 0.005); a moderate effect size was found (Cohen’s *d* = 0.50).

### Correlations between moral courage and ethical and academic factors

A Spearman correlation analysis was conducted to examine the relationships between moral courage and the variables satisfaction with the program, academic performance, relationship with educators, assessment of ethics teaching, and degree of confidence in one’s own ethical principles (Table 3).

**Table 3:** Analysis of Spearman’s correlation between moral courage and satisfaction with the program, academic performance, relationship with educators, assessment of ethics teaching, and confidence in one’s own ethical principles (N=130), Rio Grande, Rio Grande do Sul, Brazil, 2024

	Satisfaction w/ the program	Academic performance	Relationship w/ educators	Ethics teaching	Confidence in one’s won ethical principles	Moral courage
Satisfaction w/ the program	-					
Academic performance	.311^^**^^	-				
Relationship w/ educators	.376^^**^^	.456^^**^^	-			
Ethics teaching	.319^^**^^	.100	.341^^**^^	-		
Confidence in one’s won ethical principles	.217^^*^^	.145	.261^^**^^	.282^^**^^	-	
Moral courage	.188^^*^^	.297^^**^^	.231^^**^^	.115	.453^^**^^	-

* ^*^Correlation is significant at the 0.01 level (2-tailed);

* Correlation is significant at the 0.05 level (2-tailed).

Moral courage showed a weak positive correlation with satisfaction with the program (Spearman’s rho = 0.188, p = 0.032), academic performance (Spearman’s rho = 0.297, *p* = 0.001), and relationship with educator (Spearman’s rho = 0.231, *p* = 0.08), as well as a moderate positive correlation with the degree of confidence in one’s own ethical principles (Spearman’s rho = 0.446, *p* < 0.001). No significant correlation was found between moral courage and the assessment of ethics teaching (Spearman’s rho = 0.115, *p* = 0.192).

### Ethical and academic factors influencing the level of moral courage

A multiple linear regression analysis (enter method) was conducted to assess the extent to which satisfaction with the program, academic performance, relationship with educators, and degree of confidence in one’s own ethical principles influenced the level of moral courage.

The variables significantly influenced the level of moral courage, explaining 26.9% of its variance (*F*(4, 125) = 12.842, *p* < 0.001; R^2^_adjusted_ = 0.269). Table 4 presents the coefficients for all predictors. The strongest predictor of moral courage was confidence in one’s own ethical principles (*β* = 0.455, *p* < 0.001). In contrast, satisfaction with the program (*β* = 0.048, *p* > 0.05) and relationship with educators (β = -0.039, p > 0.05) had no significant impact.

**Table 4:** Predictive variables of the level of Moral Courage (N=130), Rio Grande, Rio Grande do Sul, Brazil, 2024

Predictors	Standardized coefficients		t	Sig.
	B	Beta		
(Constant)	-	-	4.985	0.000
Satisfaction w/ the program	0.546	0.048	0.565	0.573
Academic performance	2.895	0.225	2.632	0.010
Relationship with educators	-0.489	-0.039	-0.429	0.669
Confidence in one’s own ethical principles	5.706	0.455	5.678	0.000

## DISCUSSION

This is the first study to examine moral courage in Brazilian nursing, reporting a mean moral courage score of 61.58 (on a scale ranging from 15 to 75) among Brazilian nursing students. This result aligns with previous studies in other countries, where nursing students demonstrated high levels of moral courage^(16,17)^. A study addressing nursing students from six European countries found a self-assessed moral courage level of 77.80 (on a scale of 0 to 100), with significant differences between countries^(17)^. In Iran, two separate studies reported varying results: one found a mean of 52.75 (on a scale of 15 to 75)^(18)^, while a more recent study reported a mean of 58.74 (on a scale of 15 to 105); the latter indicates a moderate level of moral courage^(19)^.

These differences may be explained by cultural factors and variations in ethics education across countries^(18)^; however, social desirability bias^(20)^ may also have influenced students’ responses, as nurses are generally expected to uphold ethical standards. Consequently, nursing students may feel compelled to demonstrate moral courage and respond accordingly. Moreover, challenges in understanding the concept of moral courage-given its complexity and abstraction^(14)^-may have contributed to the high self-assessments observed.

Students in more advanced semesters exhibited higher levels of moral courage. Similarly, in Iran, moral courage was associated with educational level^(18)^. More experienced students are more likely to encounter situations requiring moral courage and tend to develop higher levels of it^(17)^, which aligns with the results of this study, in which students who had witnessed a morally incorrect or unethical situation scored higher in moral courage than those who had not.

This finding may also be linked to the phenomena of moral distress and moral resilience. Students are believed to experience moral distress when confronted with morally incorrect or unethical situations^(7)^, which necessitates moral courage^(9)^ and, consequently, moral resilience^(21)^. Studies have identified a positive and significant relationship between moral resilience and moral courage^(21,22)^, with high levels of moral courage being essential for developing moral resilience^(21)^. Thus, the more frequently students encounter situations that provoke moral distress, the more likely they are to develop moral courage and resilience.

This study also found that students with a religion exhibited higher levels of moral courage than those without one. Previous research has shown that religious orientation influences moral courage in nursing students^(19)^. A component of religion, psychological empowerment, may enhance moral courage^(10)^. Therefore, having a religion may positively influence moral courage by giving students greater confidence to act courageously.

Moral courage was correlated with satisfaction with the program, self-assessment of academic performance, relationship with educators, and confidence in one’s own ethical principles. In the search for factors that influence moral courage, the degree of confidence in one’s own ethical principles and academic performance was found to predict moral courage levels. A study corroborates these findings as it identified moral courage to be significantly associated with students deeming their academic achievements to be excellent and having greater confidence in their ethical principles^(17)^. However, the same study did not establish a relationship between moral courage and satisfaction with the nursing program. Additionally, the authors of the previously mentioned study noted inconsistency regarding this aspect, as they found a relationship between moral courage and dissatisfaction with the nursing profession^(17)^. Thus, a more in-depth analysis is required to investigate this matter.

The relevance of ethical education and the role of educators in the ethical training of nursing students is highlighted. Establishing a student/mentor relationship of mutual respect enables students to gain confidence to act when witnessing inappropriate practices. The opposite is also true, as a negative relationship between student and mentor may lead students to remain silent and not question unethical situations^(23)^. This assertion is believed to be valid in both the theoretical and practical contexts of academic training.

It is worth noting the identification of three distinct moral courage profiles among nursing students - moderate, low, and high - which highlights the heterogeneity of this attribute within the academic setting. These profiles differed significantly in terms of professional knowledge, technical skills, and perceptions of the ethical climate in clinical placements, although no significant variation was observed in moral resilience. Furthermore, students with higher moral sensitivity were more likely to belong to the moderate or high moral courage groups. These findings emphasize the relevance of understanding how diverse moral courage profiles manifest during nursing education, providing a foundation for pedagogical and institutional strategies aimed at strengthening this essential ethical competence^(24)^.

Moral courage is widely recognized as an important virtue. A bibliometric review has indicated that researchers have explored various dimensions of moral courage in nursing, including its definition and influencing factors. The countries with the highest scientific output on the topic are the United States, Finland, and China. Therefore, developing countries have not stood out in this research area, which underscores the importance of conducting studies in developing nations, such as Brazil^(25)^.

Our findings offer valuable insights into moral courage within the Brazilian context, a developing country in Latin America. It is recommended that ethics education be restructured to prioritize the cultivation of students’ ethical and moral competencies, with particular attention to moral courage and its influencing factors. Moral courage empowers students to effectively navigate ethical dilemmas when confronted with moral distress. Therefore, nursing students must develop the skills necessary to identify, critically analyze, and respond appropriately to complex and challenging situations-competencies that can be enhanced through comprehensive undergraduate nursing curricula.

Some of this study’s results are inconsistent with the literature. For example, no differences were found between age and previous training in the health field. Koskinen and collaborators^(18)^ identified higher levels of moral courage among older students with a previous degree in the health field, though these findings may be related to the specific characteristics of the sample addressed here.

Previous studies^(26,27)^ support our recommendations, highlighting that understanding the various factors influencing moral courage in nursing is crucial for addressing ethical dilemmas and enhancing patient care quality. Furthermore, the implementation of tailored educational strategies is vital to foster moral courage among nursing professionals worldwide, emphasizing the need for ongoing research and targeted interventions to strengthen ethical practice and improve care outcomes.

### Study Limitations

This study presents some limitations, including variables rated on a Likert scale in an instrument not previously validated, which does not ensure the reliability of responses. However, having experts in research ethics to assess and review the instrument mitigated this issue. Additionally, the sample size limits the generalization of results, and caution must be taken. It is worth noting that the lack of Brazilian and Latin American literature on the subject prevented more accurate comparisons. Nonetheless, we note the novelty of this study and the evidence corroborated by the international literature. Future research is suggested to seek confirmation of the predictive analyses, preferably using more robust methodological designs, such as mixed methods and longitudinal studies, and larger samples.

### Contributions to nursing

This study makes a significant contribution to the field of Nursing by demonstrating, through robust statistical analyses, the levels of moral courage among Brazilian nursing students and the factors influencing this fundamental ethical competence. By identifying ethical and academic factors associated with and predictive of moral courage in students, the findings provide valuable support for the development of pedagogical and institutional strategies aimed at strengthening ethical education. These results can guide educators, academic administrators, and policymakers in creating academic environments that foster the development of nursing professionals equipped to face complex ethical dilemmas and deliver care with ethics, critical thinking, and responsibility.

## CONCLUSIONS

This study revealed the levels of moral courage among Brazilian nursing students and the factors influencing moral courage. Such findings are particularly relevant because evidence on this topic comes mostly from developed countries, and not all the ethical and academic factors related to moral courage are known. High levels of moral courage were found among Brazilian nursing students, with students attending more advanced semesters scoring higher, as well as religious students and those who had already witnessed a morally incorrect or unethical situation.

A correlation was found between moral courage and satisfaction with the program, relationship with educators, self-assessment of academic performance, and degree of confidence in one’s own ethical principles. The factors that substantially influenced moral courage were the degree of confidence in one’s own ethical principles and academic performance. Thus, discussions between educators and students are encouraged, which could lead to interventions. Apparently, changes in the academic environment can promote moral courage and should aim to improve student satisfaction with the program and the relationship between educators and students.

Students’ confidence in their ethical principles should be strengthened alongside a program designed to enhance academic performance. Although moral courage levels were high, it should be further emphasized and critically discussed during nursing education, as ethics is fundamental to nursing care and directly impacts patient safety and quality of care.

## Data Availability

The research data are available only upon request.
