# Supplementary material for: Ethical and academic factors influencing the moral courage of nursing students
Source: Rev Bras Enferm. 2026 Aug 21;79:e20250389. doi: 10.1590/0034-7167-2025-0389 (PMC13505559; doi:10.1590/0034-7167-2025-0389)
Supplement: Translation

# Translation: Fatores éticos e acadêmicos que influenciam a coragem moral de estudantes de enfermagem

**DOI:** 10.1590/0034-7167-2025-0389pt

**Authors:** Romario Daniel Jantara, Jamila Geri Tomaschewski Barlem, Danubia Andressa da Silva Stigger, Sabrina Santos da Rocha, Rhaissa Grazielli Vieira de Azevêdo, Edison Luiz Devos Barlem, Tatiane Baratieri, Kátia Pereira de Borba

**Affiliations:** I Universidade Estadual do Centro-Oeste. Guarapuava, Paraná, Brasil; II Universidade Federal do Rio Grande. Rio Grande, Rio Grande do Sul, Brasil

**Keywords:** Coragem, Moral, Ética em Enfermagem, Enfermagem, Estudantes de Enfermagem.

## Abstract

**Objetivos:**

avaliar os níveis de coragem moral entre estudantes de graduação em enfermagem brasileiros e examinar os fatores éticos e acadêmicos que a influenciam.

**Métodos:**

estudo transversal com 130 estudantes de enfermagem, que responderam a um questionário *online* contendo informações sociodemográficas, variáveis de contexto ético e acadêmico e a versão brasileira da *Nurses’ Moral Courage Scale*. Empregou-se análises descritivas, testes t, ANOVA, correlação de Spearman e regressão linear múltipla.

**Resultados:**

a pontuação média de coragem moral foi de 61,58 (±9,55), apresentando correlação positiva com satisfação com o curso, desempenho acadêmico, relacionamento com os docentes e, principalmente, com confiança nos próprios princípios éticos. Foram preditores mais fortes a confiança nos princípios éticos (β = 0,455) e desempenho acadêmico (β = 0,225), explicando 26,9% da variância.

**Conclusões:**

Fomentar a confiança ética e o sucesso acadêmico dos estudantes pode fortalecer sua coragem moral, com implicações para a formação e desenvolvimento profissional da enfermagem.

## INTRODUÇÃO

Os profissionais de saúde enfrentam desafios éticos complexos que contribuem para o esgotamento profissional e o sofrimento moral^(1)^. O sofrimento moral é um problema reconhecido globalmente que impacta esses profissionais de forma significativa^(2)^. Entre esses profissionais, os enfermeiros desempenham um papel central no processo saúde-doença, prestando cuidados à população ao longo do ciclo de vida. Essa responsabilidade exige que esses profissionais atendam as necessidades de indivíduos com variadas patologias ou lesões, expondo os enfermeiros a estressores relevantes. Tais cenários frequentemente envolvem dilemas éticos que demandam respostas eficazes guiadas por princípios éticos e morais^(3)^.

Os desafios morais influenciam o desenvolvimento da identidade profissional e do compromisso ético dos enfermeiros, uma vez que a identidade é moldada pela autopercepção, pela função profissional, pelas relações interpessoais, pela organização do trabalho e pelas experiências^(4)^. A literatura indica que os enfermeiros muitas vezes reconhecem o curso de ação apropriado, mas frequentemente não conseguem agir de acordo com o mesmo^(5)^. Portanto, os enfermeiros devem desenvolver competências morais para lidar com essas dificuldades, a fim de fornecer cuidados de alta qualidade e mitigar o sofrimento moral^(6)^.

Os estudantes de enfermagem estão imersos nesse contexto e, em um futuro próximo, se depararão com situações eticamente desafiadoras e com sofrimento moral na sua prática profissional. Portanto, devem ser eticamente informados e moralmente sensíveis para lidar com tais situações de forma eficaz, garantindo um cuidado holístico guiado por sólida competência na tomada de decisão ética. O comportamento antiético entre os estudantes de enfermagem pode prejudicar a sua aprendizagem e formação, levando a condutas não profissionais, negligência, relações fragilizadas com os pacientes, cuidados de enfermagem abaixo do padrão e ao aumento de riscos para a segurança do paciente^(7)^.

Nesse contexto, a enfermagem é reconhecida como uma prática ética que exige coragem para defender princípios morais, advogar pelo que é certo e agir de acordo com os próprios valores. A coragem moral é essencial para os enfermeiros em todas as áreas e níveis de cuidados de saúde. O renovado interesse na ética da virtude nos cuidados de saúde contribuiu para um crescente reconhecimento da coragem moral como uma virtude e como um componente fundamental da moralidade humana^(8)^.

A coragem moral refere-se à disposição interna de um indivíduo para lidar com conflitos éticos, atuando de acordo com princípios éticos, valores pessoais e crenças, mesmo quando isso pode resultar em consequências adversas^(9)^. É uma virtude que requer deliberação e ação ética e é considerada fundamental para a competência moral geral dos enfermeiros. Profundamente ligada aos valores profissionais da enfermagem, a coragem moral é essencial para defender esses princípios na prática diária. Não se limita a atos heroicos de enfermeiros excepcionais em situações extraordinárias; trata-se de um conceito complexo e fundamental no cuidado ético de enfermagem^(8)^.

A coragem moral tem recebido crescente atenção como um componente-chave da competência moral dos enfermeiros, destacando a necessidade de identificar os fatores que a influenciam com vistas a melhorar a qualidade dos cuidados de enfermagem. Como uma virtude que pode ser ensinada, aprendida e praticada, compreender os seus determinantes, tanto a partir de perspectivas organizacionais como pessoais, é essencial para o fortalecimento ainda maior da coragem moral^(10)^.

Uma revisão de escopo identificou diversos fatores associados à coragem moral em estudantes de enfermagem, incluindo sofrimento moral, sensibilidade moral, idade e formação prévia na área da saúde, entre outros. A literatura também sugere que coragem moral pode ser um potencial preditor de transtornos de ansiedade, transtorno de estresse agudo e outras condições de saúde mental, enfatizando a necessidade de mais estudos para confirmar tais achados^(11)^. Além disso, ainda não há evidências sistemáticas e consistentes sobre os fatores éticos e acadêmicos que influenciam a coragem moral entre estudantes de enfermagem, o que é crucial para o desenvolvimento de intervenções eficazes para fortalecer a coragem moral nessa população.

## OBJETIVOS

Avaliar os níveis de coragem moral entre estudantes de graduação em enfermagem no Brasil e examinar os fatores éticos e acadêmicos que a influenciam.

## MÉTODOS

### Considerações Éticas

Este estudo seguiu os princípios éticos vigentes e cumpriu a Resolução nº 510, de 7 de abril de 2016, do Conselho Nacional de Saúde. O estudo integra um projeto maior, financiado pelo CNPq, intitulado “Sofrimento Moral, Resiliência Moral e Coragem Moral na formação e prática de enfermeiros”. O Comitê de Ética em Pesquisa aprovou o estudo por meio do parecer nº 6.034.053. O termo de consentimento livre e esclarecido foi apresentado na primeira página do formulário, enfatizando a confidencialidade das informações dos participantes, o caráter voluntário da participação, o direito de desistir do estudo a qualquer momento e a disponibilidade de assistência médica em caso de qualquer desconforto decorrente da participação.

### Delineamento, Período e Local do Estudo

Este estudo quantitativo empregou um delineamento transversal, descritivo e correlacional, sendo conduzido de acordo com as diretrizes STROBE (*Strengthening the Reporting of Observational Studies in Epidemiology*).

Os dados foram coletados entre abril e setembro de 2024 por meio de um formulário eletrônico hospedado no *Google Forms*. A autorização para convidar os alunos a participar, seja durante as aulas ou por meio de *WhatsApp* ou *e-mail*, foi obtida junto aos coordenadores e professores do curso de graduação em enfermagem. Os participantes foram informados sobre os objetivos do estudo, assegurados quanto à confidencialidade de suas informações e orientados sobre o direito de desistir do estudo a qualquer momento. Os participantes receberam um *link* para acessar o formulário eletrônico, que ficou disponível após a assinatura de consentimento livre e esclarecido, apresentado na primeira página. Uma cópia eletrônica do formulário e das respostas foi enviada automaticamente para o e-mail cadastrado dos participantes. Sempre que possível, o tempo de aula foi reservado para que os alunos preenchessem o questionário, que exigia aproximadamente 20 minutos.

### População e Amostra: Critérios de Inclusão e Exclusão

Foi utilizada amostragem por conveniência não probabilística, com o tamanho da amostra calculado utilizando o módulo *StatCalc* do Epi Info 7.2.5.0^(12)^. O critério de inclusão exigia que os participantes estivessem regularmente matriculados a partir do terceiro semestre, visto que era necessária experiência clínica prática para o preenchimento do questionário. O tamanho mínimo da amostra necessário, considerando os 186 estudantes de enfermagem matriculados a partir do terceiro semestre na instituição de ensino participante, foi determinado em 125 estudantes, considerando um nível de confiança de 95%. Ao final, 130 estudantes de enfermagem completaram o formulário do estudo, sem desistências ou respostas faltantes.

### Protocolo do Estudo

Foi desenvolvido um questionário sociodemográfico para caracterizar as variáveis do contexto ético e acadêmico, e adotou-se a versão brasileira da *Nurses’ Moral Courage Scale - NMCS* ^(13)^.

O questionário sociodemográfico e a caracterização das variáveis do contexto ético e acadêmico foram divididos em duas seções: (1) Dados Sociodemográficos e (2) Formação Profissional e Acadêmica. O formulário continha 33 itens para a coleta de variáveis sociais, sociodemográficas e contextuais que podem influenciar a coragem moral, conforme identificado na literatura internacional^(11)^. Estado civil, etnia, renda familiar, religião e semestre do curso foram respondidos por meio de opções predefinidas. A idade foi medida em anos, enquanto o sexo foi categorizado dicotomicamente (masculino/feminino). Além disso, as seguintes variáveis foram avaliadas como dicotômicas (sim/não): formação prévia na área da saúde, ter presenciado uma situação moralmente incorreta ou antiética envolvendo um colega e participa ativamente de uma atividade relacionada à ética (por exemplo, em um grupo de pesquisa).

As variáveis de contexto ético e acadêmico incluíram satisfação com o curso, autoavaliação do desempenho acadêmico, avaliação do relacionamento com os docentes, avaliação do ensino de ética no curso e confiança nos próprios princípios éticos. Esses itens foram avaliados em escala *Likert,* e doutores com experiência em ética em enfermagem revisaram o instrumento.

A Escala de Coragem Moral dos Enfermeiros foi originalmente desenvolvida na Finlândia. Inicialmente, era composta por 21 itens distribuídos em quatro dimensões: (1) compaixão e presença verdadeira, (2) responsabilidade moral, (3) integridade moral e (4) compromisso com o bom atendimento. As respostas são avaliadas em uma escala Likert de 5 pontos, na qual os respondentes indicam o quanto cada item os descreve, variando de 1 (não me descreve de forma alguma) a 5 (me descreve muito bem). Pontuações mais altas refletem maior coragem moral autoavaliada^(14)^.

A escala foi validada no contexto brasileiro para ser usada com estudantes de enfermagem, adotando uma estrutura unidimensional com 15 itens e uma faixa de pontuação de 15 a 75. A escala demonstrou fortes índices de confiabilidade, com o Limite Inferior Maior de Confiabilidade em 1,00, Ômega de McDonald em 0,91 e Alfa de Cronbach em 0,91^(13)^.

### Análise dos dados e estatística

Os dados foram tabulados no Microsoft Excel e analisados quantitativamente utilizando estatística descritiva e inferencial. A análise descritiva incluiu cálculos de frequências, médias, desvio padrão, assimetria e curtose. A estatística inferencial compreendeu o teste t de *Student*, ANOVA de um fator, a correlação de Spearman e a regressão linear múltipla. O pacote estatístico SPSS, versão 20.0, foi utilizado em todas as análises.

A normalidade dos escores de Coragem Moral foi avaliada utilizando o teste de Kolmogorov-Smirnov com correção de Lilliefors e o teste de Shapiro-Wilk. Os resultados indicaram que a variável Coragem Moral não apresentou distribuição normal (K-S(130) = 0,094, p = 0,006; S-W(130) = 0,906, p < 0,001), sendo o mesmo padrão observado para as demais variáveis.

A ANOVA foi realizada para examinar as diferenças nas médias de coragem moral em relação ao estado civil, semestre do curso, faixa etária, renda familiar, etnia e religião. O teste t de Student foi utilizado para comparar as médias de coragem moral entre os grupos com base em sexo, reprovação em alguma disciplina, formação prévia na área da saúde, ter presenciado um evento moralmente incorreto ou antiético e ter participado ativamente em atividades relacionadas à ética. Além disso, o teste t foi empregado para comparar as médias de coragem moral entre os estudantes agrupados do terceiro ao sexto semestre e do sétimo ao décimo semestre, assim como entre os estudantes com e sem religião.

A correlação de Spearman foi utilizada para examinar a relação entre coragem moral e variáveis contextuais éticas e acadêmicas, incluindo satisfação com o curso, desempenho acadêmico, relacionamento com docentes, avaliação do ensino de ética e confiança nos próprios princípios éticos. Uma análise de regressão linear múltipla (método *enter* do *software* SPSS) foi conduzida para identificar preditores de coragem moral, satisfação com o curso, desempenho acadêmico, relacionamento com docentes e confiança nos próprios princípios éticos como variáveis independentes.

A homogeneidade da variância foi avaliada utilizando o teste de Levene para orientar a interpretação dos testes estatísticos. O método de *bootstrap* com 1.000 reamostragens e um intervalo de confiança de 95% com correção de viés e aceleração (BCa) foi aplicado para lidar com desvios da normalidade e aumentar a confiabilidade dos resultados. Esse procedimento permite estimar que, se o estudo fosse replicado 100 vezes, os resultados estariam dentro da margem de erro em 95% dos casos^(15)^.

## RESULTADOS

### Caracterização sociodemográfica

A maioria dos 130 participantes era do sexo feminino (86,2%), com idade média de 24,64 anos (DP = 5,19; variação: 19-48). A maioria era solteira (84,6%), se autodeclarava branca (74,6%), tinha renda familiar entre uma e duas vezes o salário mínimo (44,6%) e relatou não ter religião (38,5%). Em relação aos semestres do curso, 16,9% estavam no terceiro semestre, 14,6% no quarto, 16,2% no quinto, 10,8% no sexto, 16,9% no sétimo, 8,5% no oitavo, 14,6% no nono e 1,5% no décimo semestre.

### Estatísticas descritivas da coragem moral e outras variáveis

A pontuação total variou de 17 a 75 pontos, com média de 61,58 e desvio padrão de 9,55. A Tabela 1 apresenta os resultados descritivos das demais variáveis.

**Tabela 1:** Resultados descritivos referentes ao semestre do curso, ensino de ética e sofrimento moral entre estudantes de enfermagem (N=130), Rio Grande, Rio Grande do Sul, Brasil, 2024

Variáveis		N=130	Porcentagem (%)
Semestre do curso	3^º^ semestre	22	16,9
	4^º^ semestre	19	14,6
	5^º^ semestre	21	16,2
	6^º^ semestre	14	10,8
	7^º^ semestre	22	16,9
	8^º^ semestre	11	8,5
	9^º^ semestre	19	14,6
	10^º^ semestre	2	1,5
Reprovação no curso	Sim	45	34,6
	Não	85	65,4
Formação anterior na área da saúde	Sim	23	17,7
	Não	107	82,3
Presenciou um evento moralmente incorreto ou antiético envolvendo um colega de classe	Sim	60	46,2
	Não	70	53,8
Participa ativamente em uma atividade relacionada à ética (por ex.: grupo de pesquisa)	Sim	12	9,2
	Não	118	90,8
Frequência que sente que deveria agir com coragem moral	Média (DP^*^)		4,19 (DP=0,93)
Nível de satisfação com o curso	Média (DP^*^)		3,63 (DP = 0,83)
Autoavaliação do desempenho acadêmico	Média (DP^*^)		3,74 (DP = 0,74)
Avaliação do relacionamento com os docentes	Média (DP^*^)		3,82 (DP = 0,75)
Avaliação do ensino de ética no curso	Média (DP^*^)		3,59 (DP =1,00)
Confiança nos próprios princípios éticos	Média (DP^*^)		4,20 (DP =0,76)

### Diferenças nos escores médios de coragem moral de acordo com as características sociodemográficas da amostra

A ANOVA corrigida para dados sem homogeneidade de variância, conforme indicado pelo teste de Levene (Levene 2, 127) = 3,171, p = 0,045) não identificou diferenças significativas nas médias de coragem moral entre estudantes solteiros, casados ou em união estável (F de Welch (2, 12,034) = 0,353, p = 0,710). Da mesma forma, nenhuma diferença estatisticamente significativa foi encontrada entre os semestres do curso (F(7, 122) = 1,511, p = 0,170) (Levene(7, 122) = 0,568, p = 0,780). No entanto, o teste t de Student revelou que, quando os estudantes foram agrupados entre aqueles que cursavam do terceiro ao sexto semestre e aqueles que cursavam do sétimo ao décimo semestre, estes últimos apresentaram escores mais altos de coragem moral (Tabela 2). Foi identificado um tamanho de efeito pequeno (d de Cohen = 0,37).

**Tabela 2:** Resultados do teste de diferença nos escores de coragem moral entre os grupos de semestres: teste t (análise com bootstrap) (N=130), Rio Grande, Rio Grande do Sul, Brasil, 2024

		*M*	*DP*	*t*	*gl*	Diferença média	IC da diferença média (95%)	
							Limite inferior	Limite superior
Coragem Moral	3^º^ ao 6^º^	60,15	10,03	-2,08^^*^^	128	-3,50	57,98	62,26
	7^º^ ao 10º	63,66	8,48				61,34	65,81

M - média; DP - desvio padrão;

* p - 0,039; gl - graus de liberdade; IC - intervalo de confiança.

A ANOVA também não mostrou diferença estatisticamente significativa entre os níveis de coragem moral dos estudantes nas diferentes faixas etárias (20 a 24 anos, 25 a 29 anos e acima de 29 anos) (Levene (2, 127) = 0,496, p = 0,496; F (2, 127) = 0,460, p = 0,632), diferentes rendas familiares (Levene (3, 126) = 1,705, p = 0,169; F (3, 126) = 0,621, p = 0,603), etnias (Levene (3, 126) = 0,530, p = 0,663; F (3, 126) = 0,819, p = 0,486) ou entre diferentes religiões e ausência de religião (Levene (4, 125) = 0,611, p = 0,655; F (4, 125) = 1,518, p = 0,201). No entanto, quando analisados em dois grupos, o teste t de Student mostrou que participantes com religião exibiram escores estatisticamente mais altos de coragem moral (M = 62,97; DP = 8,84) do que aqueles sem religião (M = 59,36; DP = 10,29) (t(128) = -2,127, p = 0,035).

Não foram encontradas diferenças significativas nos escores médios de coragem moral com base no sexo (t(128) = 0,917, p = 0,361), reprovação no curso (t(128) = 1,114, p = 0,267), formação prévia na área da saúde (t(128) = -0,562, p = 0,575) ou participação ativa em uma tarefa relacionada à ética (t(128) = 0,410, p = 0,682). No entanto, os estudantes que presenciaram uma situação moralmente incorreta ou antiética apresentaram escores significativamente mais altos em coragem moral (M = 64,11, DP = 7,72) do que aqueles que não presenciaram (M = 59,41, DP = 10,45) (t(128) = 2,874, p = 0,005); com um tamanho de efeito moderado (d de Cohen = 0,50).

### Correlações entre coragem moral e fatores éticos e acadêmicos

Foi realizada uma análise de correlação de Spearman para examinar as relações entre a coragem moral e as variáveis satisfação com o curso, desempenho acadêmico, relacionamento com os docentes, avaliação do ensino de ética e grau de confiança nos próprios princípios éticos (Tabela 3).

**Tabela 3:** Análise de correlação de Spearman entre coragem moral e satisfação com o curso, desempenho acadêmico, relacionamento com docentes, avaliação do ensino de ética e confiança nos próprios princípios éticos (N=130), Rio Grande, Rio Grande do Sul, Brasil, 2024

	Satisfação com o curso	Desempenho acadêmico	Relacionamento com docentes	Ensino de ética	Confiança nos próprios princípios éticos	Coragem moral
Satisfação com o curso	-					
Desempenho acadêmico	,311^^**^^	-				
Relacionamento com docentes	,376^^**^^	,456^^**^^	-			
Ensino de ética	,319^^**^^	,100	,341^^**^^	-		
Confiança nos próprios princípios éticos	,217^^*^^	,145	,261^^**^^	,282^^**^^	-	
Coragem moral	,188^^*^^	,297^^**^^	,231^^**^^	,115	,453^^**^^	-

* ^*^Correlação é significante ao nível 0,01 (teste bicaudal);

* Correlação é significante ao nível 0,05 (teste bicaudal).

A coragem moral apresentou uma correlação positiva fraca com a satisfação com o curso (rho de Spearman = 0,188, p = 0,032), desempenho acadêmico (rho de Spearman = 0,297, p = 0,001) e relacionamento com docentes (rho de Spearman = 0,231, p = 0,08), bem como uma correlação positiva moderada com o grau de confiança nos próprios princípios éticos (rho de Spearman = 0,446, p < 0,001). Não foi encontrada correlação significativa entre coragem moral e a avaliação do ensino de ética (rho de Spearman = 0,115, p = 0,192).

### Fatores éticos e acadêmicos que influenciam o nível de coragem moral

Foi realizada uma análise de regressão linear múltipla pelo método *enter* do *software* SPSS para avaliar em que medida a satisfação com o curso, o desempenho acadêmico, o relacionamento com os docentes e o grau de confiança nos próprios princípios éticos influenciaram o nível de coragem moral.

As variáveis influenciaram significativamente o nível de coragem moral, explicando 26,9% da sua variância (F(4, 125) = 12,842, p < 0,001; R^2^_ajustado_ = 0,269). A Tabela 4 apresenta os coeficientes para todos os preditores. O preditor mais forte de coragem moral foi a confiança nos próprios princípios éticos (β = 0,455, p < 0,001). Em contrapartida, a satisfação com o curso (β = 0,048, p > 0,05) e o relacionamento com os docentes (β = -0,039, p > 0,05) não tiveram impacto significativo.

**Tabela 4:** Variáveis preditivas do nível de coragem moral (N=130), Rio Grande, Rio Grande do Sul, Brasil, 2024

Preditores	Coeficientes padronizados		t	Sig.
	B	Beta		
(Constante)	-	-	4,985	0,000
Satisfação com o curso	0,546	0,048	0,565	0,573
Desempenho acadêmico	2,895	0,225	2,632	0,010
Relacionamento com docentes	-0,489	-0,039	-0,429	0,669
Confiança nos próprios princípios éticos	5,706	0,455	5,678	0,000

## DISCUSSÃO

Este é o primeiro estudo a examinar a coragem moral na enfermagem brasileira, relatando um escore médio de coragem moral de 61,58 (em uma escala de 15 a 75) entre estudantes de enfermagem brasileiros. Esse resultado está em consonância com estudos realizados em outros países, nos quais estudantes de enfermagem demonstraram altos níveis de coragem moral^(16,17)^. Um estudo com estudantes de enfermagem de seis países europeus encontrou um nível de coragem moral autoavaliado de 77,80 (em uma escala de 0 a 100), com diferenças significativas entre os países^(17)^. No Irã, dois estudos distintos relataram resultados diferentes: um encontrou uma média de 52,75 (em uma escala de 15 a 75)^(18)^, enquanto um estudo mais recente relatou uma média de 58,74 (em uma escala de 15 a 105); este último resultado indica um nível moderado de coragem moral^(19)^.

Essas diferenças podem ser explicadas por fatores culturais e variações na educação em ética entre os países^(18)^; no entanto, o viés de desejabilidade social^(20)^ também pode ter influenciado as respostas dos estudantes, uma vez que geralmente se espera que os enfermeiros defendam padrões éticos. Assim, os estudantes de enfermagem podem sentir-se compelidos a demonstrar coragem moral e a responder de acordo. Além disso, as dificuldades em compreender o conceito de coragem moral - dada a sua complexidade e abstração^(14)^- podem ter contribuído para as elevadas autoavaliações observadas.

Alunos em semestres mais avançados demonstraram níveis mais elevados de coragem moral. Da mesma forma, no Irã, a coragem moral foi associada ao nível educacional^(18)^. Alunos mais experientes têm maior probabilidade de se deparar com situações que exigem coragem moral e tendem a desenvolver níveis mais elevados dessa característica^(17)^, o que está em consonância com os resultados deste estudo, segundo os quais os estudantes que presenciaram uma situação moralmente incorreta ou antiética obtiveram escores mais altos em coragem moral do que aqueles que não a presenciaram.

Essa descoberta também pode estar relacionada aos fenômenos de sofrimento moral e resiliência moral. Acredita-se que os alunos vivenciem sofrimento moral quando confrontados com situações moralmente incorretas ou antiéticas^(7)^, o que exige coragem moral^(9)^ e, consequentemente, resiliência moral^(21)^. Estudos identificaram uma relação positiva e significativa entre resiliência moral e coragem moral^(21,22)^, sendo que altos níveis de coragem moral são essenciais para o desenvolvimento da resiliência moral^(21)^. Assim, quanto mais frequentemente os alunos se depararem com situações que provocam sofrimento moral, maior é a probabilidade de desenvolverem coragem moral e resiliência.

Este estudo também constatou que os estudantes com religião apresentaram níveis mais elevados de coragem moral do que aqueles sem religião. Estudos anteriores demonstraram que a orientação religiosa influencia a coragem moral em estudantes de enfermagem^(19)^. Um componente da religião, o empoderamento psicológico, pode aumentar a coragem moral^(10)^. Portanto, a religiosidade pode influenciar positivamente a coragem moral, conferindo aos estudantes maior confiança para agir de forma eticamente corajosa.

A coragem moral correlacionou-se com a satisfação com o curso, a autoavaliação do desempenho acadêmico, o relacionamento com docentes e a confiança nos próprios princípios éticos. Na análise dos fatores associados à coragem moral, o grau de confiança nos próprios princípios éticos e o desempenho acadêmico foram identificados como preditores dos níveis de coragem moral. Um estudo corrobora esses achados, ao identificar que a coragem moral está significativamente associada à percepção dos alunos de que suas conquistas acadêmicas são excelentes e à maior confiança em seus princípios éticos^(17)^. Contudo, o mesmo estudo não estabeleceu associação entre coragem moral e satisfação com o curso de enfermagem. Além disso, os autores do estudo mencionado acima observaram inconsistência em relação a esse aspecto, pois encontraram uma relação entre coragem moral e insatisfação com a profissão de enfermagem^(17)^. Portanto, análises mais aprofundadas são necessárias para investigar essa questão.

Destaca-se a relevância da educação ética e o papel dos professores na formação ética de estudantes de enfermagem. O estabelecimento de uma relação de respeito mútuo entre alunos e mentor permite que os alunos adquiram confiança para agir ao presenciarem práticas inadequadas. O oposto também é verdadeiro, pois uma relação negativa entre aluno e mentor pode levar os alunos a permanecerem em silêncio e não questionarem situações antiéticas^(23)^. Acredita-se que essa afirmação seja válida tanto no contexto teórico quanto no contexto prático da formação acadêmica.

Vale ressaltar a identificação de três perfis distintos de coragem moral entre os estudantes de enfermagem - baixo, moderado e alto - o que destaca a heterogeneidade desse atributo no ambiente acadêmico. Esses perfis evidenciaram diferenças significativas em termos de conhecimento profissional, habilidades técnicas e percepções do clima ético nos estágios clínicos, embora nenhuma variação significativa tenha sido observada quanto à resiliência moral. Além disso, os estudantes com maior sensibilidade moral apresentaram maior probabilidade de pertencer aos grupos de coragem moral moderada ou alta. Esses achados enfatizam a relevância de compreender como os diversos perfis de coragem moral se manifestam durante a formação em enfermagem, fornecendo uma base para estratégias pedagógicas e institucionais voltadas ao fortalecimento dessa competência ética essencial^(24)^.

A coragem moral é amplamente reconhecida como uma virtude importante. Uma revisão bibliométrica indicou que os pesquisadores exploraram várias dimensões da coragem moral na enfermagem, incluindo sua definição e fatores de influência. Os países com maior produção científica sobre o tema são os Estados Unidos, Finlândia e China. Assim, os países em desenvolvimento não se destacaram nessa área de pesquisa, o que ressalta a importância de conduzir estudos nesses contextos, como o brasileiro^(25)^.

Esses resultados oferecem informações valiosas sobre a coragem moral no contexto brasileiro, um país da América Latina ainda em desenvolvimento. Recomenda-se que o ensino da ética seja reestruturado para priorizar o desenvolvimento das competências éticas e morais dos estudantes, com atenção especial à coragem moral e aos seus fatores de influência. A coragem moral capacita os alunos a lidar de forma eficaz com dilemas éticos quando confrontados com sofrimento moral. Portanto, os estudantes de enfermagem devem desenvolver as habilidades necessárias para identificar, analisar criticamente e responder adequadamente a situações complexas e desafiadoras - competências que podem ser aprimoradas por meio de currículos abrangentes de graduação em enfermagem.

Alguns dos resultados deste estudo divergem da literatura. Por exemplo, não foram encontradas diferenças em relação à idade nem à formação prévia na área da saúde. Koskinen e colaboradores^(18)^ identificaram níveis mais elevados de coragem moral entre estudantes mais velhos e entre aqueles com formação prévia na área da saúde, embora esses resultados possam estar relacionados às características específicas da amostra do presente estudo.

Estudos anteriores^(26,27)^ corroboram nossas recomendações, destacando que a compreensão dos diversos fatores que influenciam a coragem moral na enfermagem é crucial para lidar com dilemas éticos e aprimorar a qualidade da atenção ao paciente. Além disso, a implementação de estratégias educacionais personalizadas é vital para fomentar a coragem moral entre os profissionais de enfermagem em todo o mundo, enfatizando a necessidade de pesquisa contínua e de intervenções direcionadas para fortalecer a prática ética e melhorar os resultados da atenção à saúde.

### Limitações do estudo

Este estudo apresenta algumas limitações, incluindo a utilização de variáveis avaliadas em escala *Likert* em um instrumento não previamente validado, o que pode comprometer a confiabilidade das respostas. No entanto, a participação de especialistas em ética em enfermagem para avaliar e revisar o instrumento contribuiu para mitigar esse problema. Além disso, o tamanho da amostra limita a generalização dos resultados, exigindo cautela em sua interpretação. Cabe ressaltar que a escassez de literatura brasileira e latino-americana sobre o tema restringiu comparações mais precisas. Não obstante, destaca-se a originalidade deste estudo e a consonância dos achados com a literatura internacional. Sugere-se que pesquisas futuras busquem confirmar as análises preditivas, preferencialmente utilizando delineamentos metodológicos mais robustos, como métodos mistos e estudos longitudinais, além de amostras de maior porte.

### Contribuições para enfermagem

Este estudo contribui significativamente para a área da enfermagem ao demonstrar, por meio de análises estatísticas robustas, os níveis de coragem moral entre estudantes de enfermagem brasileiros e os fatores que influenciam essa competência ética fundamental. Ao identificar fatores éticos e acadêmicos associados e preditores da coragem moral entre estudantes, os resultados fornecem suporte relevante para o desenvolvimento de estratégias pedagógicas e institucionais voltadas ao fortalecimento da educação em ética. Esses resultados podem orientar professores, gestores acadêmicos e formuladores de políticas educacionais na criação de ambientes acadêmicos que promovam o desenvolvimento de profissionais de enfermagem capacitados para enfrentar dilemas éticos complexos e prestar cuidados baseados em ética, pensamento crítico e responsabilidade profissional.

## CONCLUSÕES

Este estudo revelou os níveis de coragem moral entre estudantes brasileiros de enfermagem e os fatores que a influenciam. Tais achados são particularmente relevantes porque as evidências sobre o tema provêm, em sua maioria, de países desenvolvidos, e ainda não são plenamente conhecidos todos os fatores éticos e acadêmicos relacionados à coragem moral. Foram identificados altos níveis de coragem moral entre os estudantes de enfermagem brasileiros, sendo que aqueles em semestres mais avançados apresentaram escores mais altos, assim como os estudantes com religião e aqueles que haviam presenciado uma situação moralmente incorreta ou antiética.

Foi encontrada uma correlação entre coragem moral e satisfação com o curso, relacionamento com docentes, autoavaliação do desempenho acadêmico e grau de confiança nos próprios princípios éticos. Portanto, os fatores que influenciaram de forma mais substancial a coragem moral foram o grau de confiança nos próprios princípios éticos e o desempenho acadêmico. O diálogo entre professores e estudantes deve ser encorajado pois pode levar ao desenvolvimento de intervenções. Mudanças no ambiente acadêmico podem promover a coragem moral e devem visar à melhoria da satisfação dos estudantes com o curso, assim como ao fortalecimento do relacionamento entre docentes e estudantes.

A confiança dos estudantes em seus princípios éticos deve ser fortalecida por meio de estratégias educacionais voltadas à melhoria do desempenho acadêmico. Embora os níveis de coragem moral tenham se mostrado elevados, esse aspecto deve ser ainda mais enfatizado e discutido criticamente durante a formação em enfermagem, visto que a ética é fundamental para o cuidado de enfermagem e impacta diretamente a segurança do paciente e a qualidade da assistência.

## Data Availability

Os dados de pesquisa só estão disponíveis mediante solicitação.
